# Cardioproteção durante a Quimioterapia: Perspectivas de Estratégias com Antioxidantes

**DOI:** 10.36660/abc.20210914

**Published:** 2021-11-22

**Authors:** Thiago Gomes Heck

**Affiliations:** 1 Universidade Regional do Noroeste do Estado do Rio Grande do Sul Programa de Pós-Graduação em Modelagem Matemática e Computacional Ijuí RS Brasil Grupo de Pesquisa em Fisiologia, Programa de Pós-Graduação em Atenção Integral à Saúde (PPGAIS), Programa de Pós-Graduação em Modelagem Matemática e Computacional (PPGMMC), Universidade Regional do Noroeste do Estado do Rio Grande do Sul (UNIJUÍ), Ijuí, RS – Brasil

**Keywords:** Cardioproteção, Tratamento Farmacológico, Antioxidantes, Estresse Oxidativo, Resveratrol

O conceito de estresse oxidativo, bem definido como uma situação em que células, tecidos ou todo o organismo apresentam um desequilíbrio entre as espécies reativas de oxigênio (EROS) e as defesas antioxidantes em favor dos níveis de EROS, se apresenta em diversas doenças agudas e crônicas, como o câncer. O equilíbrio entre a produção de EROS e a capacidade das células de evitar o dano oxidativo é um desafio natural para as espécies de mamíferos, uma vez que a produção de energia pelas vias oxidativas nas mitocôndrias permite a produção contínua de radicais livres e moléculas oxidativas.^1^ Os agentes farmacológicos, em geral, podem usar a modulação de equilíbrio oxidativo para tratar algumas doenças, ou o desequilíbrio oxidativo pode representar um efeito colateral dos medicamentos. Além disso, precisamos ter em mente que o estresse oxidativo causado por condições ambientais (como, por exemplo, poluição do ar) e metabólicas (como, por exemplo, obesidade e diabetes) também está associado a maior risco de diversos cânceres.^2^

A hiperproliferação de células tumorais é acompanhada por alta produção de EROS, mas sabe-se que a resposta ao estresse celular, as defesas antioxidantes e a resistência contra os efeitos citotóxicos da quimioterapia são bastante diferentes no tumor em comparação com as células do sistema imunológico e outros tecidos. Assim, o tumor é capaz de se adaptar às condições de carga oxidativa, enquanto outras células podem não apresentar autodefesa suficiente contra o dano oxidativo. O mecanismo de autoproteção tumoral envolve aumento dos níveis da principal defesa antioxidante não enzimática, a glutationa (GSH).^2^ Como uma célula “faminta”, considerando a demanda comum por altos níveis de energia principalmente pela captação de glicose, os tumores ativam a glicose-6-fosfato desidrogenase (G6PD) e redireciona o metabolismo da glicose desde a glicólise até o braço oxidativo da via das pentoses-fosfato (VPF) em direção à síntese de nucleotídeos. Por sua vez, o aumento da nicotinamida adenina dinucleotídeo fosfato (NADPH) permite o aumento da GSH e de outros sistemas antioxidantes. Além disso, em nível molecular, o tumor evoca diversos fatores de transcrição, incluindo a proteína ativadora 1 (AP-1), fator de choque térmico 1 (HSF1), fator nuclear κB (NF-κB), fator nuclear eritroide 2 relacionado ao fator 2-p45 (NRF2), e proteína tumoral p53, representando uma estratégia proliferativa acompanhada por uma defesa robusta contra o estresse celular.^2,3^

Em meio a essa tempestade, é necessário atacar o tumor com agentes agressivos. As antraciclinas, como a doxorrubicina (DOX), são altamente eficazes contra leucemias linfoblásticas e mieloblásticas agudas e também contra tumores sólidos (como, por exemplo, câncer de mama). No entanto, a cardiotoxicidade induzida pelas antraciclina pode ocorrer de forma aguda (durante o tratamento ou imediatamente após), induzindo pericardite-miocardite ou arritmias, promovendo complicações crônicas e aumentando o risco de morte. No coração, por ser um órgão com intenso metabolismo oxidativo por natureza, a DOX pode aumentar a produção de EROS ao metabolizar a droga. Enzimas mitocondriais, como a NADPH oxidase, citocromo P-450 redutase e xantina oxidase, podem transformar a DOX e outras antraciclinas na forma de quinona em semiquinona, que por sua vez gera EROS, como o radical ânion superóxido, entre outros. Além disso, EROS derivadas de DOX podem ativar o fator p53 e a sinalização pró-inflamatória, como vias centradas em NF-κB, resultando em um desequilíbrio entre proteínas pró e anti-apoptóticas e anti-inflamatórias. Por fim, a DOX pode afetar a transcrição e a expressão de proteínas cardíacas específicas, prejudicando a função dos cardiomiócitos, levando à deterioração miofibrilar, interrupção da organização do sarcômero e redução da função contrátil.^4^

Evitando o cenário oxidativo, a busca básica é por estratégias antioxidantes. Nesta edição, o estudo^5^ mostrou que a suplementação com resveratrol durante o período gestacional tem efeito cardioprotetor no coração dos filhotes contra a toxicidade induzida pela DOX. Esse tratamento antioxidante induziu um aumento na viabilidade celular dos cardiomiócitos neonatais e diminuição dos marcadores apoptóticos/necróticos. Esses efeitos estiveram associados à melhora na defesa antioxidante e diminuição dos danos oxidativos ao DNA, e promoveram a expressão de proteínas relacionadas às vias de resposta celular ao estresse (Sirt6) nos cardiomiócitos dos filhotes. Além disso, esses resultados animadores mostraram a relevância da suplementação com antioxidantes durante o tratamento do câncer e destacaram a relevância de incluir intervenções relacionadas aos antioxidantes (por exemplo: exercícios)^6,7^ em ambientes maternos em resposta a agentes de estresse para a saúde dos filhotes a fim de evitar a cardiotoxicidade.
