# Supplementary material for: Cardioproteção durante a Quimioterapia: Perspectivas de Estratégias com Antioxidantes
Source: Arq Bras Cardiol. 2021 Nov 22;117(6):1159–60. [Article in Portuguese] doi: 10.36660/abc.20210914 (PMC8757159; doi:10.36660/abc.20210914)
Supplement: Translation

# Translation: Cardioprotection during Chemotherapy: Prospects of Antioxidant Strategies

**Authors:** Thiago Gomes Heck

**Affiliations:** 1 Ijuí RS Brazil Grupo de Pesquisa em Fisiologia, Programa de Pós-Graduação em Atenção Integral à Saúde (PPGAIS), Programa de Pós-Graduação em Modelagem Matemática e Computacional (PPGMMC), Universidade Regional do Noroeste do Estado do Rio Grande do Sul (UNIJUÍ), Ijuí, RS – Brazil

**Keywords:** Cardioprotection, Drug Therapy, Antioxidants, Oxidative Stress, Resveratrol

The concept of oxidative stress, well defined as a situation in which cells, tissues, or the whole body present an imbalance between reactive oxygen species (ROS) and antioxidant defenses in favor of ROS levels, is found in several acute and chronic diseases, including cancer. The equilibrium between ROS production and the ability of cells to avoid oxidative damage is a natural challenge for mammalian species since the energy production by oxidative pathways in mitochondria allows the continuous production of free radical and oxidative molecules.^1^ Pharmacological agents may generally use the modulation of oxidative balance to treat some diseases, or oxidative imbalance may represent a side effect of the drugs. Also, we need to keep in mind that oxidative stress caused by environmental (e.g., air pollution) and metabolic (e.g., obesity and diabetes) conditions is also associated with an increased risk of a variety of cancers.^2^

Hyperproliferation of tumor cells is accompanied by high ROS production, but it is well known that cell stress response, antioxidant defenses, and resistance against the cytotoxic effects of chemotherapy is quite different in tumor compared to immune cells and other tissues. Thus, the tumor is able to adapt to conditions of oxidative burden while other cells may not present sufficient self-defense against oxidative damage. The mechanism of tumor self-protection involves increasing the levels of the major non-enzymatic antioxidant defense, glutathione (GSH).^2^ As a “hungry” cell, considering the common high-energy demand mainly by glucose uptake, tumors activate glucose-6-phosphate dehydrogenase (G6PD) and reroute glucose metabolism from glycolysis through the oxidative arm of the pentose phosphate pathway (PPP) toward nucleotide synthesis. In turn, the increase in nicotinamide adenine dinucleotide phosphate (NADPH) allows the increase in GSH and other antioxidant systems. Also, at a molecular level, the tumor evocates many transcription factors, including activator protein 1 (AP-1), heat shock factor 1 (HSF1), nuclear factor κB (NF-κB), nuclear factor-erythroid 2 p45-related factor 2 (NRF2), and tumor protein p53, representing a proliferative strategy accompanied by a robust cell stress defense.^2,3^

In the middle of this storm, it is necessary to attack the tumor with aggressive agents. Anthracyclines, such as doxorubicin (DOX), are highly effective against acute lymphoblastic and myeloblastic leukemias and also against solid tumors (e.g., breast cancer). However, anthracycline-induced cardiotoxicity may occur acutely (during treatment or immediately after), inducing pericarditis-myocarditis or arrhythmias, chronically promoting complications, and increasing the risk of death. In the heart, as an organ with an intense oxidative metabolism by nature, DOX can increase ROS production by metabolizing the drug. Mitochondria enzymes such as NADPH oxidase, cytochrome P-450 reductase, and xanthine oxidase can transform DOX and other anthracyclines in the form of quinone into semiquinone, which in turn generates ROS as superoxide anion and others. Also, DOX-derived ROS may activate p53 and pro-inflammatory signaling such as NF-κB-centered pathways, resulting in an imbalance between pro-and anti-apoptotic and anti-inflammatory proteins. Finally, DOX could affect the transcription and expression of cardiac-specific proteins impairing the function of cardiomyocytes leading to myofibrillar deterioration, disruption of sarcomere organization, and reduction of contractile function.^4^

Avoiding the oxidative scenario, the basic search is for antioxidant strategies. In this issue, a study^5^ showed that supplementation with resveratrol during the gestational period has a cardioprotective effect on the offspring's heart against DOX-induced toxicity. This antioxidant treatment induced an increase in neonatal cardiomyocyte cell viability and decreased apoptotic/necrotic markers. These effects were associated with improved antioxidant defense and decreased DNA oxidative damages, and promoted the expression of proteins related to cell stress response pathways (Sirt6) in the cardiomyocytes of the pups. Furthermore, these exciting results showed the relevance of antioxidant supplementation during cancer treatment and highlighted the relevance of including antioxidant-related interventions (e.g. exercise)^6,7^ in maternal environments in response to stress agents for the offspring health to avoid cardiotoxicity.
